# Editorial: Genomic discoveries and pharmaceutical development in urologic tumors

**DOI:** 10.3389/fphar.2024.1508979

**Published:** 2024-12-09

**Authors:** Jialin Meng, Huan Yang, Lei Yin

**Affiliations:** ^1^ Department of Urology, The First Affiliated Hospital of Anhui Medical University, Hefei, China; ^2^ Institute of Urology and Anhui Province Key Laboratory of Urological and Andrological Diseases Research and Medical Transformation, Anhui Medical University, Hefei, China; ^3^ School of Life Sciences, Anhui Medical University, Hefei, China; ^4^ Department of Urology, Tongji Hospital, Tongji Medical College, Huazhong University of Science and Technology, Wuhan, China; ^5^ Department of Urology, Ruijin Hospital, Shanghai Jiao Tong University School of Medicine, Shanghai, China; ^6^ Department of Urology, Shanghai Ninth People’s Hospital, Shanghai Jiao Tong University School of Medicine, Shanghai, China

**Keywords:** urologic tumors, cancer biomarkers, drug screen, drug repurposing, genomic sequence

The prevalence of urologic tumors is rapidly increasing, leading to severe clinical outcomes. In the United States, approximately 169,360 new cases of urologic tumors are estimated for 2024, with about 32,350 tumor-specific deaths (Siegel et al., 2024). Similarly, China faces a staggering annual estimate of 244,300 new cases, resulting in 113,700 deaths (Han et al., 2024). These alarming statistics underscore the urgent need for advanced therapeutic strategies and personalized treatments to improve survival rates for patients suffering from urologic tumors.

A particularly exciting area of research is genomic sequencing, which facilitates the identification of specific genetic mutations and biomarkers for targeted drug therapies. This includes recognizing molecular subtypes that enable precision clinical therapy (Li et al., 2024; Figiel et al., 2024). For instance, drugs targeting the androgen receptor (AR) pathway, such as abiraterone and enzalutamide, have demonstrated efficacy in treating prostate cancers with relevant genetic alterations (Dai et al., 2023). Additionally, drugs like sunitinib and pazopanib, which inhibit the vascular endothelial growth factor (VEGF) pathway, have significantly improved survival rates in patients with metastatic renal cell carcinoma (George et al., 2019). This Research Topic explores cutting-edge therapies, molecular markers, and mechanisms reshaping the field.

Advanced-stage castration-resistant prostate cancer presents significant challenges in clinical practice, often necessitating combination therapy strategies. Consequently, the search for novel clinical treatments, the exploration of tumor progression signaling pathways, and the identification of prognostic biomarkers are imperative. In this Research Topic, Wei et al. report that apalutamide neoadjuvant therapy enhances resectability in unresectable prostate cancer, facilitating successful surgeries. Zhang et al. recommend cabazitaxel plus prednisone as the most effective first-line treatment for metastatic castration-resistant prostate cancer. Meanwhile, Lampe et al. discuss challenges associated with bi-specific T-cell engagers (BiTEs) therapy for prostate cancer and suggest improvement strategies. In terms of mechanisms, Zhang et al. review the role of m6A regulators in prostate cancer, particularly in AR signaling pathways and disease progression. Chen et al. demonstrate that combining PTEN restoration with IL-23 inhibition significantly enhances therapeutic outcomes in metastatic prostate cancer. Lin et al. discover that SGLT2 inhibition reduces prostate cancer risk by modulating circulating metabolites, especially uridine levels. Finally, Xia et al. identify anoikis-related gene signatures as potential prognostic markers for prostate cancer bone metastasis.

Urothelial bladder cancer exhibits one of the highest mutation burdens among all cancer types (Cancer Genome Atlas Research, 2014), contributing to variable patient responses to immunotherapy, particularly with the use of *bacillus* Calmette-Guérin for early-stage non-muscle invasive bladder cancer (Morales, 1992). In this Research Topic, Peng et al. find that IL4I1 expression in bladder cancer correlates with better responses to immune checkpoint inhibitors. Zhu et al. show that HER2 positivity in urothelial carcinoma is associated with advanced disease stages and combining disitamab vedotin with PD-1 inhibitors shows promise. Uysal et al. reveal that EGFR, AREG, and EREG amplification predicts poor survival in muscle-invasive bladder cancer. Additionally, Li et al. suggest that rosuvastatin may reduce bladder cancer risk, while other statins appear ineffective.

Furthermore, Geng et al. develop a vasculogenic mimicry gene signature that predicts survival and immune responses in clear cell renal cell carcinoma. Huang et al. identify GJA5 and GJB1 as prognostic markers for renal clear cell carcinoma, with lower expression linked to poorer outcomes. Song et al. show that KHSRP knockdown inhibits the progression of papillary renal cell carcinoma and enhances gemcitabine sensitivity. Qu et al. report a rare case of ossifying renal tumor of infancy, emphasizing the need for careful monitoring due to high Ki-67 expression. Duan et al. explore alternative splicing events associated with clinical features across multiple cancers, offering insights into tumor progression and immune infiltration.

This Research Topic highlights advances in genomic discoveries and therapeutic approaches. While significant progress has been made in understanding the molecular mechanisms and targeted therapies for conditions like advanced-stage castration-resistant prostate cancer and metastatic renal cell carcinoma, challenges remain, particularly regarding resistance to existing therapies and the necessity for personalized treatment options. The studies presented here underscore the importance of integrating novel biomarkers and therapeutic strategies, such as BiTEs and m6A regulators, to enhance patient outcomes. Future research should prioritize several key areas. First, deeper investigations into the interactions between the tumor microenvironment and the immune system are essential for elucidating the mechanisms of immune evasion by tumors, which could pave the way for novel immunotherapies. Furthermore, identifying and validating new therapeutic targets, especially those linked to resistance mechanisms, will be crucial for improving patient prognoses. The integration of genomics, proteomics, and metabolomics data to create more comprehensive personalized treatment strategies could significantly enhance therapeutic outcomes for patients.

In conclusion, while substantial strides have been made in the research and treatment of urologic tumors, ongoing efforts are necessary. Through interdisciplinary collaboration and innovation, integrating new technologies and discoveries, we can significantly improve the prognosis and quality of life for patients with urologic tumors. The vision for the future includes not only better therapeutic strategies but also a more profound understanding of cancer biology, ultimately leading to effective treatments tailored to individual patient needs. We eagerly anticipate the application of these research findings in clinical practice, which could revolutionize treatment options for urologic tumor patients.
